# Synthetic Protein Scaffolds Based on Peptide Motifs and Cognate Adaptor Domains for Improving Metabolic Productivity

**DOI:** 10.3389/fbioe.2015.00191

**Published:** 2015-11-23

**Authors:** Anselm H. C. Horn, Heinrich Sticht

**Affiliations:** ^1^Bioinformatik, Institut für Biochemie, Friedrich-Alexander-Universität Erlangen-Nürnberg, Erlangen, Germany

**Keywords:** adaptor domain, linear peptide motif, protein scaffold, fusion protein, metabolic engineering

## Abstract

The efficiency of many cellular processes relies on the defined interaction among different proteins within the same metabolic or signaling pathway. Consequently, a spatial colocalization of functionally interacting proteins has frequently emerged during evolution. This concept has been adapted within the synthetic biology community for the purpose of creating artificial scaffolds. A recent advancement of this concept is the use of peptide motifs and their cognate adaptor domains. SH2, SH3, GBD, and PDZ domains have been used most often in research studies to date. The approach has been successfully applied to the synthesis of a variety of target molecules including catechin, D-glucaric acid, H_2_, hydrochinone, resveratrol, butyrate, gamma-aminobutyric acid, and mevalonate. Increased production levels of up to 77-fold have been observed compared to non-scaffolded systems. A recent extension of this concept is the creation of a covalent linkage between peptide motifs and adaptor domains, which leads to a more stable association of the scaffolded systems and thus bears the potential to further enhance metabolic productivity.

## Introduction

Nature has developed highly efficient ways for signal and substrate processing in living cells. Synthetic biology is inspired by the natural archetype and tries to mimic and optimize biological processes for tailor-made applications (Luo et al., 2013). Milestone achievements of this relatively young area include the microbial production of artemisinic acid, a key precursor of the antimalarial drug artemisinin (Martin et al., 2003; Ro et al., 2006), the industrial production of 1,3-propanediol, used in a multitude of further applications (Nakamura and Whited, 2003), and the reconstruction of a complete microbial genome (Gibson et al., 2010).

The rational design in synthetic biology is frequently inspired by the spatial proximity of enzymes observed in nature (Conrado et al., 2008; Luo et al., 2013). Following evolution, such artificial bioreactors implement proximity mostly via modular scaffolds (Carroll, 2005; Bhattacharyya et al., 2006). In this concept, modular building blocks are used for the creation of large custom scaffold systems. Such scaffolds define the spatial organization of enzymes and allow substrate channeling like in natural systems, which has several advantages: it rescues the intermediates from diffusion or competing pathways, decreases their transit times, and avoids unfavorable equilibria and kinetics from metabolite concentrations in the bulk phase (Miles et al., 1999; Spivey and Ovadi, 1999). In nature, many organisms have developed multifunctional enzyme systems with a pivotal role in both primary metabolism [e.g., amino acid biosynthesis (Welch and Gaertner, 1980) or fatty acid oxidation (Ishikawa et al., 2004)] and secondary metabolism [e.g., multifunctional polyketide synthases in bacteria (Pfeifer and Khosla, 2001) and flavonoid or alkaloid biosynthesis in plants (Jorgensen et al., 2005)].

Multifunctional enzyme systems that mimic natural systems can be artificially constructed via at least four general strategies: (I) *colocalization* or *immobilization* of enzymes has been the first approach to be of practical use; (II) *compartmentalization* generates an enclosed reaction area that can be defined similar to biological systems (e.g., in cell organelles); (III) *DNA/RNA building blocks* can be utilized for spatial organization of reactive centers; and (IV) *protein scaffolding* presents a versatile approach in synthetic biology. This last scaffolding principle can be divided further into several approaches: fusion proteins are constructed by linking two or more enzymes into a single protein sequence. Non-covalent protein–protein interactions via the mutual recognition of folded domains or coiled-coil pairs as well as amyloid assemblies can be used to construct scaffolds with defined stoichiometry. A brief overview over all these scaffolding strategies is provided as supplementary information. Another concept in protein-based scaffolding uses protein adaptor domains and peptide ligands for bringing enzymes in spatial proximity, the focus of this review.

## Scaffolding Based on Adaptor Domains: Structural Principles

**Figure 1:**
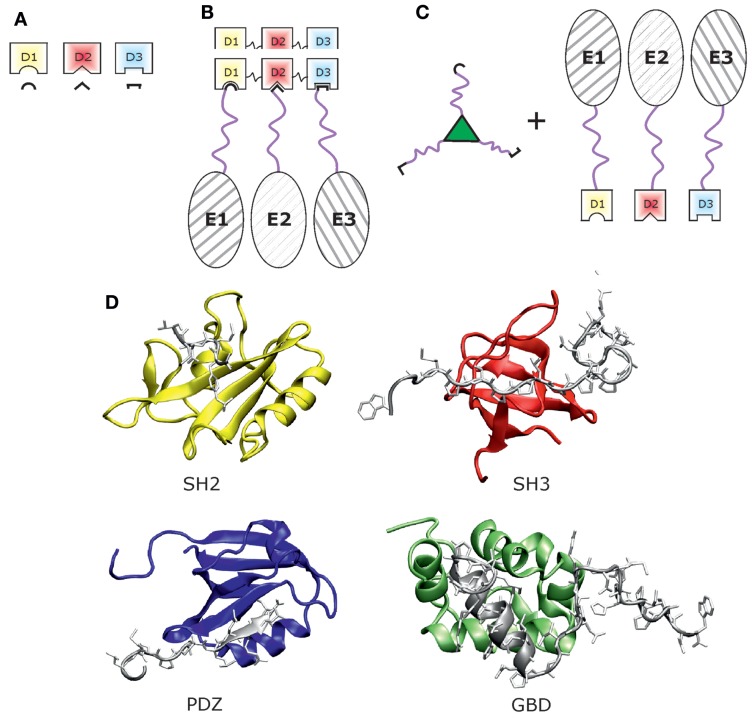
**Scaffolding with adaptor domains and peptide motifs**. **(A)** Schematic view of scaffolding modules, i.e., three different adaptor domains (D1–D3) with their peptide ligands (black line forms). **(B)** Scaffold protein built by the three adaptor domains D1–D3 with three enzymes (E1–E3) bound via peptide ligands, which are fused by a linker region (pink) to the respective enzyme. **(C)** Alternative scaffold system formed by three peptide ligands [cf. Lu et al. (2014)], which bind to the respective adaptor domain fused to an enzyme. **(D)** Three-dimensional structures of protein domains used for scaffolding: SH2 domain in yellow [PDB-code: 3WA4, Higo et al. (2013)], SH3 domain in red [PDB-code: 1WA7, Schweimer et al. (2002)], PDZ domain in blue [PDB-code: 4UU5, Ivanova et al. (2015)], and GBD domain in green [PDB-code: 2K42, Cheng et al. (2008)]. Structural representations were created with VMD (Humphrey et al., 1996).

Many important physiological protein interactions are mediated by relatively small protein domains, which bind to peptides exhibiting specific sequence motifs (Figure 1A) (Dinkel and Sticht, 2010). In this type of interaction, only the adaptor domain adopts a globular three-dimensional structure while the interaction motif is mostly linear and has, therefore, been termed short linear interaction motif (SLiM). This type of protein–ligand interaction presents a promising concept in protein scaffolding (Figure 1B) that has gained a lot of attention in synthetic biology applications.

Protein scaffolding based on cognate adaptor domains and peptide motifs requires a careful selection of candidate domains and SLiMs as well as the choice of proper linkers to interconnect these moieties and to attach them to the enzymes of interest (Figure 1B). The properties of these three building blocks, i.e., domain, linker, and peptide ligand, critically affect the shape of the resulting scaffold (cf. Figure 1) and will be described in the following in more detail.

### Adaptor Domains

Adaptor domains used in protein-peptide scaffolding need to fulfill two basic requirements. First, they should have a strong affinity toward their peptide ligands to allow for effective coupling. Second, they should provide a distinct specificity for their ligands to allow for defined coupling, when several domain-ligand pairs are used simultaneously. The most often used domains are SH3, SH2, PDZ, and GTPase-binding domain (GBD) (cf. Figure 1D).

The first protein modules that were reported to mediate interaction with SLiMs are the “Src homology 2” (SH2) and “Src homology 3” (SH3) domains (Koch et al., 1991). SH3 domains are small modules of ca. 60 residues. They recruit proline-rich ligands, which bind to the domain surface at three shallow grooves formed by conserved aromatic residues (Mayer, 2001) and exhibit two different binding orientations. Over the last few years, an increasing number of SH3 domains with different ligand binding specificity have been described (Saksela and Permi, 2012).

SH2 domains are highly conserved structures of ca. 100 residues comprising two α-helices and seven β-strands (Pawson et al., 2001). In nature, this domain possesses an either promiscuous or strict specificity for a 3–5 residues motif flanking a phosphorylated tyrosine; like for the SH3 domain, additional SH2-binding modes were discovered, underscoring the plasticity of this recognition type in physiological context (Machida and Mayer, 2005).

PDZ domains are also widely used for scaffolding. They are of similar size as SH2 domains and target specific motifs at the C-terminus of the binding partner. The peptide ligand adopts a β-strand and extends an existing β-sheet within the PDZ domain upon binding (Schultz et al., 1998; Harris and Lim, 2001). At least four different classes of ligands are known for PDZ domains exhibiting a distinct binding specificity (Songyang et al., 1997).

The last example of an established domain-ligand pair in synthetic biology originates from GBDs. In contrast to the other domains discussed above, isolated GBD domains do not adopt a single, discrete structure under physiological conditions but rather sample multiple, loosely packed conformations in solution (Abdul-Manan et al., 1999; Kim et al., 2000). The corresponding peptide ligand has been deduced from the autoinhibited form of the GBD (Dueber et al., 2009). Figure 1D shows three-dimensional structures of the SH2, SH3, PDZ, and GBD domain. Beyond the examples presented above, other domain/ligand pairs may also be utilized for synthetic scaffolds if they exhibit a sufficiently high affinity and specificity for their ligand.

### Linear Motif Peptides

Short linear interaction motifs are the complementary binding partner to protein adaptor domains. These peptide motifs occur in disordered protein regions and are present in 20–50% of all eukaryotic proteins, while up to 17% of the proteins are completely disordered in eukaryotic cells. To date, ~300 known motif patterns are listed in electronic databases, e.g., ELM database (Dinkel et al., 2014), PROSITE (Hulo et al., 2004), and Minimotif-Miner (Balla et al., 2006). Interestingly, there are estimates that in the proteome the SLiM-mediated instances in signaling pathway modulation outnumber those mediated by globular domains (McEntyre and Gibson, 2004).

Linear motif peptides possess a number of properties, which make them well suited as ligands in synthetic biology. The interaction motifs normally comprise only 3–10 amino acids and are thus rather short and intrinsically disordered. Furthermore, SLIMs may constitute the sites of post-translational modification (e.g., phosphorylation), which enables them to function as inducible switches.

In addition to the key residues necessary for binding, SLiMs also frequently contain variable residues (denoted as “X”) to ensure proper spacing between the binding residues. Due to its lack of a defined structure prior to binding, this peptide–domain interaction differs from the well-known domain–domain interactions in protein complexes. Prominent examples for SLiM sequence patterns include the classical P–x–x–P motif for binding to SH3 domains or a phosphorylated tyrosine with specific sequence neighbors for binding to SH2 domains.

### Linkers

The last part necessary for modular protein scaffolding is the linker region connecting the engineered enzymes and the attached peptide ligands (Figure 1B). The importance of linker design is well known from fusion proteins (Chen et al., 2013), as length and amino acid composition may influence the activity and folding properties of the protein construct (Robinson and Sauer, 1998; Bai and Shen, 2006; Zhao et al., 2008).

As a guide for the rational design of artificial linkers, an inspection of natural linkers is helpful. Two independent studies with different data sets gave similar results: while Argos found a preferred mean linker length of 6.5 residues (Argos, 1990), George and Heringa obtained a value of 10.0 ± 5.8 residues (George and Heringa, 2002). Generally, polar or charged residues were enriched in the natural linkers, with a secondary structure preference for coil (Argos, 1990) or helix (George and Heringa, 2002), respectively. Natural linkers lack interaction with neighboring protein domains and adopt mainly non-globular conformations (Chen et al., 2013).

Designed linkers may be classified according to their structure, which defines their functionality. Flexible linkers are normally rich in small or hydrophilic amino acids and allow for an increased spatial separation and reorientation of the fused parts. A prominent and very early example for a flexible linker is (GGGGS)_3_ that connected the heavy and light chain domains (V_H_ and V_L_) of an engineered antibody fragment (Huston et al., 1988). Rigid linkers with the sequence (EAAAK)*_n_* exhibit a stable helical structure and thus pertain a certain distance between the fused parts. This linker type has been successfully used to increase the enzymatic efficiency of bifunctional fusions of β-glucanase and xylanase (Lu and Feng, 2008).

It should also be noted that linker regions may also have additional benefits. They potentially improve folding and stability (Huston et al., 1988; Takamatsu et al., 1990; Werner et al., 2006; Hagemeyer et al., 2009), expression (Amet et al., 2009), or even bioactivity (Bai and Shen, 2006).

## Scaffolding Based on Adaptor Domains: Application to Metabolic Engineering

In this section, several applications of scaffolding using adaptor domains and peptide ligands are presented. The key features of the engineered systems are summarized in Table 1.

**Table 1:** **Examples of engineered scaffolds comprising adaptor domains and peptide ligands**.

Product	Enzyme pathway^a^	Domains^b^	Host	Fold increase^c^	Reference
Mevalonate	Acetoacetyl-CoA thiolase, hydroxy-methylglutaryl-CoA synthase, hydroxymethylglutaryl-CoA reductase	GBD, SH3, PDZ	*E. coli*	77	Dueber et al. (2009)
D-glucaric acid	Myo-inositol-1-phosphate synthase, myo-inositol oxygenase, (uronate dehydrogenase)	(GBD), SH3, PDZ	*E. coli*	3	Dueber et al. (2009)
D-glucaric acid	Myo-inositol-1-phosphate synthase, myo-inositol oxygenase, uronate dehydrogenase	GBD, SH3, PDZ	*E. coli*	5	Moon et al. (2010)
H_2_	[Fe-Fe]-hydrogenase, ferredoxin, (pyruvate-ferredoxin oxidoreductase)	(GBD), SH3, PDZ	*E. coli*	3–5	Agapakis et al. (2010)
Hydrochinone	Cutinase	SH2	Self-assembled monolayer	30	Li et al. (2010)
Resveratrol	4-Coumarate:CoA ligase, stilbene synthase	(GBD), SH3, PDZ	*S. cerevisiae*	5	Wang and Yu (2012)
Butyrate	(Acetoacetyl-CoA thiolase), 3-hydroxybutyryl-CoA dehydrogenase, 3-hydroxybutyryl-CoA dehydratase, trans-enoyl-coenzyme A reductase, (acyl-CoA thioesterase II)	GBD, SH3, PDZ	*E. coli*	3	Baek et al. (2013)
Gamma-aminobutyric acid	Glutamate decarboxylase, glutamate/GABA antiporter	SH3	*E. coli*	2.5	Vo et al. (2013)
Catechin	Flavanone 3-hydroxylase, dihydroflavonol 4-reductase, leucoanthocyanidin reductase	GBD, SH3, PDZ	*E. coli*	1.3	Zhao et al. (2015)

*^a^Unscaffolded enzymes in parentheses*.

*^b^Scaffold domains without a ligand/enzyme counterpart in parentheses*.

*^c^Compared to the unscaffolded system*.

As one prominent example for this approach, Dueber et al. (2009) engineered a model scaffold for the three-step synthesis of mevalonate (Martin et al., 2003), which is an important precursor for the large field of isoprenoids, starting from acetyl-CoA. The enzymatic system comprised three modules, acetoacetyl-CoA thiolase (AtoB), hydroxy-methylglutaryl-CoA synthase (HMGS) and hydroxymethylglutaryl-CoA reductase (HMGR). From these modules, only AtoB is native to the host system *Escherichia coli*, whereas the two others were imported from *Saccharomyces cerevisiae*. To avoid flux imbalances with high metabolic load and to increase the overall production, scaffold constructs of three domains, GBD, SH3, and PDZ connected via flexible linkers were created and AtoB, HMGS, and HMGR were extended by corresponding peptide ligands, respectively. As this first simple scaffold design yielded only slightly increased product titers compared to the scaffold-free system, the authors designed scaffolding proteins with a varying number of SH3 and PDZ domains. This systematic search revealed the best synthetic scaffold GBD_1_–SH3_2_–PDZ_2_ for this system, i.e., one GBD domain linked to two SH3 and PDZ domains, and exhibited a remarkable 77-fold increase of the product. Furthermore, the authors also investigated the influence of the spatial orientation of the domains toward each other by changing the order of the two SH3 and PDZ domains. A further increase, however, was not observed in these additional systems (Dueber et al., 2009).

In order to demonstrate the generality of this approach, the same group strived to increase the production of d-glucaric acid from d-glucose via scaffolding. The synthetic pathway had originally been constructed by Moon et al. (2009): myo-inositol-1-phosphate synthase (Ino1) from *S. cerevisiae*, myo-inositol oxygenase (MIOX) from mouse, and uronate dehydrogenase (Udh) from *Pseudomonas syringae* were coexpressed in *E. coli*. A domain-based scaffold for the two enzymes Ino1 and MIOX, which were equipped with the respective peptide ligand sequences, tripled the product titers compared to the original system (Dueber et al., 2009). Additional optimization of the system by including Udh into the scaffold and also varying the number of cognate domains within the scaffold allowed for an additional product increase of ~50% (Moon et al., 2010).

The first artificially scaffolded redox pathway was presented by Agapakis et al. (2010). They engineered a hydrogen-producing electron transfer circuit in *E. coli* composed of the heterologously expressed enzymes [Fe-Fe]-hydrogenase, ferredoxin, and pyruvate-ferredoxin oxidoreductase. A major issue was the risk of side reactions caused by high energy electrons stored in iron-sulfur cluster proteins. They, thus, applied several methods to insulate the synthetic pathway, one of which was to utilize a protein scaffold constructed from the three domains GBD, SH3, and PDZ. This approach yielded a threefold increase of H_2_ production. Furthermore, the authors investigated the influence of scaffold protein composition and peptide ligand linker length on the yield and found both to be a significant factor.

Scaffolds consisting of the same domains, GBD, SH3, and PDZ, were used to increase the production of butyrate in *E. coli* (Baek et al., 2013). For the complete biosynthetic pathway, the five enzymes acetoacetyl-CoA thiolase, 3-hydroxybutyryl-CoA dehydrogenase, 3-hydroxybutyryl-CoA dehydratase, trans-enoyl-coenzyme A reductase, acyl-CoA thioesterase II were overexpressed in the host. For the three enzymes amidst the pathway, a domain scaffold was created to provide a better spatial proximity of the reaction centers. After additional variation of the domain frequency within the scaffold, the production increased to threefold.

Wang and Yu (2012) used the set of scaffold proteins composed of GBD, SH3, and PDZ domains established by Dueber et al. (2009) for another biotechnological application. Their work aimed to recruit two enzymes, 4-coumarate:CoA ligase and stilbene synthase, via covalently attached SH3 and PDZ peptide ligands for the biosynthesis of resveratrol, a naturally occurring defense molecule from plants with significant physiological effects on human and animals. In contrast to the experimental settings discussed above, *S. cerevisiae* was used as host system. The product yield increased fivefold via the scaffolding approach compared to the unscaffolded enzymes and 2.7-fold compared to a direct fusion protein approach.

The biosynthesis pathway of catechins from flavanone was the target of metabolic engineering efforts of Koffas and coworkers (Zhao et al., 2015). In their pathway optimization, they focused on three enzymes: flavanone-3-hydroxylase, dihydroflavonol 4-reductase, and leucoanthocyanidin reductase. Application of scaffolds composed of GBD, SH3, and PDZ domains yielded only marginal metabolic improvement in some cases, whereas most constructs tested exhibited a decreased productivity.

Besides the creation of a scaffold protein containing multiple adaptor domains, domain–ligand interactions can also be exploited in a different fashion as exemplified by the work of Vo et al. (2013). They enhanced the productivity of *E. coli* producing gamma-aminobutyric acid (GABA) by coupling glutamate decarboxylase (GadA/GadB) to the membrane protein glutamate/GABA antiporter (GadC). For that purpose, they attached an SH3 domain to GadA/GadB and three peptide ligand sequences to GadC each separated with flexible linkers. In that way, they could increase the GABA productivity by 2.5-fold.

A further and different approach used the localization of substrate and enzyme on a self-assembled monolayer for a 30-fold product increase (Li et al., 2010); 4-hydroxyphenyl 2-methylvalerate, which is converted by cutinase to a hydroquinone product, and a SH2-ligand were presented on the surface to the enzyme fused to a SH2 domain. As the SH2 domain only recognizes its ligand in phosphorylated form, the system contains a potential switch, which might be exploited in future applications.

## Outlook

Inspection of Table 1 reveals that the increase in metabolic productivity is highly dependent on the system investigated, and for some of the systems, there is little benefit from scaffolding. As suggested by Zhao et al. (2015), a further increase in catechin biosynthesis might be achieved from an optimization of linkers. An additional factor for optimization might be the use of alternative adaptor domains or the rational design of covalent bonds between the two binding partners in order to increase the stability of the scaffolded complex.

Recently, Lu et al. (2014) constructed two domain-ligand pairs for both SH3 and PDZ domains, in which the ligand–domain interaction was reinforced by an engineered thioether bond. For that purpose, a residue within the domain was mutated to cysteine, while the peptide ligand was equipped with an unnatural amino acid carrying a reactive α-chloroacetyl group. Binding of the ligand to the domain brought the two reactants in close proximity and established the covalent bond. Using this approach, the authors constructed several Y-shaped ligand structures via triazole bonds branched from a lysine site as mini-scaffolds (Figure 1C).

Similarly, Guan et al. (2013) created a disulfide bond between a PDZ domain and its ligand by mutating one residue to cysteine in each of the binding partners to reinforce the domain–ligand interaction. Fusing these modified moieties to the trimeric protein CutA, they were able to build stable hydrogels. By adding a second peptide ligand sequence to one CutA species, the hydrogel could be functionalized by an enzyme and formed an enzymatic biocathode for direct electron transfer.

A new versatile approach for covalent protein linkage is based on CnaB domains from bacteria, which autocatalytically establish isopeptide bonds between the sidechains of a lysine and an asparagine/aspartate residue (Veggiani et al., 2014). By a structure-based splitting of the CnaB domain into two parts, it was possible to create a domain-ligand pair that enables spontaneous formation of intermolecular isopeptide linkages (Zakeri et al., 2012; Li et al., 2014). A modification of this approach even allows that two peptides become covalently joined by an artificial ligase (Fierer et al., 2014). A recently described ester bond that forms autocatalytically in a bacterial cell surface adhesion protein (Kwon et al., 2014) also bears the potential for the construction of orthogonal covalent domain/ligand pairs. The enhanced stability due to the isopeptide or ester bonds may present a promising strategy to design more efficient scaffolds for artificial bioreactors in the future.

Synthetic biology is an emerging field with tremendous biotechnological potential. The efforts reviewed above clearly demonstrate that promising steps in this field have been made, though individual system design will require a tailor-made approach for achieving optimization. More complex metabolic pathways or large-scale industrial applications, however, would clearly benefit from an extended and well characterized tool-box of scaffolding components (Kwok, 2010).

## Conflict of Interest Statement

The authors declare that the research was conducted in the absence of any commercial or financial relationships that could be construed as a potential conflict of interest.
