# Supplementary material for: Synthetic Protein Scaffolds Based on Peptide Motifs and Cognate Adaptor Domains for Improving Metabolic Productivity
Source: Front Bioeng Biotechnol. 2015 Nov 23;3:191. doi: 10.3389/fbioe.2015.00191 (PMC4655305; doi:10.3389/fbioe.2015.00191)
Supplement: Supplementary file 1 [file Data_Sheet_1.PDF]

Supplementary Material

Synthetic protein scaffolds based on peptide motifs and cognate adaptor domains for improving metabolic productivity

Anselm H. C. Horn[1*], Heinrich Sticht[1*]

[1] Bioinformatik, Institut für Biochemie

Friedrich-Alexander-Universität Erlangen-Nürnberg (FAU), Fahrstr. 17, 91054 Erlangen, Germany

*** Correspondence:** Dr. Anselm Horn, anselm.horn@fau.de; Prof. Dr. Heinrich Sticht, heinrich.sticht@fau.de

General scaffolding concepts

To follow nature's paradigm of spatial proximity of enzymatic cascades several scaffolding concepts have been proposed and realized. In the following we provide a short overview of several scaffolding approaches, which do not rely on the interaction between peptide motifs and adaptor domains.

I. Immobilization

From a historical point of view, the first approach for colocalization of different enzymes was immobilizing enzymes on a matrix via chemical cross-linking. In 1970, Mosbach and Mattiasson constructed a two-enzyme-system from hexokinase and glucose-6-phosphate dehydrogenase that yielded an increase in product formation of up to 140% compared to the free enzyme system (Mosbach and Mattiasson, 1970). This method was later successfully extended to other two- and three-enzyme systems with varying improvement factors (Srere et al., 1973;Koch-Schmidt et al., 1977;Mansson et al., 1983). While this approach presents a proof of principle for the general concept for spatial proximity of sequential pathway enzymes, modern techniques strive for organizing such pathways in a more biological environment, at best in living cells, to account for an easier manipulation and scale-up (Conrado et al., 2008).

II. Compartmentalization

Another structural approach derived from nature is the formation of microcompartments, in which cargo proteins are enclosed. A three-dimensional compartimentalization can be achieved by virus capsid protein building blocks. Such capsid proteins self-assemble into uniform nm-scale structures. Minten et al. linked enzymes via a coiled-coil interaction to the capsid protein of Cowpea Chlorotic Mottle virus (CCMV), which forms icosahedral structures in a pH-dependent way and created a reactive compartment (Minten et al., 2011). In a similar approach, the coat protein from bacteriophage Qβ was used to construct a virus-like particle with enzyme cargo load. The authors reported successful RNA-directed encapsidation of three different enzymes (Fiedler et al., 2010).

III. DNA/RNA based scaffolding

For a more controlled spatial proximity, nucleic acid based scaffolding techniques were developed. These allow the creation of nanoscaffolds for the organization of multi-enzyme complexes (Pinheiro et al., 2011). Müller and Niemeyer conjugated glucose oxidase and horseradish peroxidase to different DNA oligonucleotide sequences, which arranged on a complementary DNA scaffolding strand. The resulting structure presented a sequential two-enzyme system working on microplates (Müller and Niemeyer, 2008). The same enzyme pair was coupled to hinges of two-dimensional DNA scaffolds, which self-organize from DNA strips in different hexagonal structures (Wilner et al., 2009). Conrado et al. used plasmid DNA as scaffold for arranging biosynthetic enzymes in the cytoplasm of *E. coli*. Individual enzymes were transformed into custom DNA-binding proteins via genetic fusion to zinc-finger domains that recognize unique DNA sequences. When expressed in cells, the scaffold architecture caused an increase of various metabolic products (Conrado et al., 2012).

For in vivo applications, RNA assemblies provide a versatile material. An engineered RNA module folded into a duplex with PP7 and MS2 aptamer domains and were arranged further into one and two-dimensional scaffolds. PP7 andMS2 fusion proteins from hydrogenase and ferredoxin bound to this scaffold exhibited up to 48-fold increase of hydrogen production compared to the unscaffolded proteins (Delebecque et al., 2011).

IV. Protein-based scaffolding concepts

In this section, we shortly present scaffolding concepts, which although based on proteins are not the main topic of this review, but are listed here for the sake of completeness. Such concepts include a direct covalent linkage as fusion proteins (see IV.1) or different types of adaptors for noncovalent linkage (IV.2 to IV.4).

IV.1 Fusion proteins

An established method for the creation of artificial multifunctional enzymes is the integration of two or more different catalytic protein domains into one molecule by covalently linking them together (Yu et al., 2015). Fusion proteins have successfully been used for protein purification (Terpe, 2003) and imaging (Yuste, 2005), as well as biopharmaceuticals (Chen et al., 2012). A remarkable application was the engineering of plant-derived cytochrome P450 enzymes into *E. coli* that allowed the robust production of plant estrogen pharmaceuticals (Leonard and Koffas, 2007). Despite its wide applicability this approach only works well for enzyme pairs whereas the fusion of more components is challenging and frequently results in aggregating or catalytically inactive enzymes (Chen et al., 2014).

IV.2 Protein-protein recognition

The high-affinity interaction between two globular proteins was also utilized for the creation of scaffolding systems. The best-documented system is derived from plant cellulosomes that degrade cellulose and cell walls. The macromolecular assembly consists of two parts, a protein scaffold, which is formed by different dockerin proteins, and enzymatic moieties each fused to a cohesin protein as distinct interaction partner for the respective dockerin within the scaffold. The resulting cellulosome chimeras exhibited enhanced synergistic action on crystalline cellulose (Fierobe et al., 2001;Mingardon et al., 2007a;Mingardon et al., 2007b). You and Zhang used three different cohesins and three dockerin-containing enzymes to assemble a complex containing triosephosphate isomerase, aldolase, and fructose-1,6-biphosphatase. This enzyme complex exhibited one order of magnitude higher initial reaction rates than a mixture of the three unscaffolded enzymes (You and Zhang, 2014).

IV.3 Coiled-coil pairs

A relatively simple protein interaction motifs is the versatile coiled-coil structure, in which peptides of ca. 20 – 50 residues adopt a helical fold and oligomerize into helix bundles of homo- or heteromers. This property has for example been used for the enzyme linkage to the CCMV capsid protein (Minten et al., 2011) described in section II. The coiled-coil toolkit has recently been expanded systematically via the SYNZIP database of heterospecific synthetic coiled-coil peptides. It includes 27 pairs of interacting peptides that prefer a specific heteroassociation (Reinke et al., 2010;Thompson et al., 2012).

IV.4 Protein lego

A relatively new concept for engineered protein proximity is the use of amyloids assemblies. Peptides, which are prone to amyloidogenic aggregation, are fused to the proteins of interest and then assemble into large scaffold structures (Giraldo, 2010).

Supplementary references

Chen, R., Chen, Q., Kim, H., Siu, K.H., Sun, Q., Tsai, S.L., and Chen, W. (2014). Biomolecular scaffolds for enhanced signaling and catalytic efficiency. *Curr Opin Biotechnol* 28**,** 59-68. doi: 10.1016/j.copbio.2013.11.007.

Chen, X., Zaro, J.L., and Shen, W.C. (2012). Pharmacokinetics of recombinant bifunctional fusion proteins. *Expert Opin Drug Metab Toxicol* 8**,** 581-595. doi: 10.1517/17425255.2012.673585.

Conrado, R.J., Varner, J.D., and Delisa, M.P. (2008). Engineering the spatial organization of metabolic enzymes: mimicking nature's synergy. *Curr Opin Biotechnol* 19**,** 492-499. doi: 10.1016/j.copbio.2008.07.006.

Conrado, R.J., Wu, G.C., Boock, J.T., Xu, H., Chen, S.Y., Lebar, T., Turnsek, J., Tomsic, N., Avbelj, M., Gaber, R., Koprivnjak, T., Mori, J., Glavnik, V., Vovk, I., Bencina, M., Hodnik, V., Anderluh, G., Dueber, J.E., Jerala, R., and Delisa, M.P. (2012). DNA-guided assembly of biosynthetic pathways promotes improved catalytic efficiency. *Nucleic Acids Research* 40**,** 1879-1889. doi: 10.1093/nar/gkr888.

Delebecque, C.J., Lindner, A.B., Silver, P.A., and Aldaye, F.A. (2011). Organization of intracellular reactions with rationally designed RNA assemblies. *Science* 333**,** 470-474. doi: 10.1126/science.1206938.

Fiedler, J.D., Brown, S.D., Lau, J.L., and Finn, M.G. (2010). RNA-directed packaging of enzymes within virus-like particles. *Angew Chem Int Ed Engl* 49**,** 9648-9651. doi: 10.1002/anie.201005243.

Fierobe, H.P., Mechaly, A., Tardif, C., Belaich, A., Lamed, R., Shoham, Y., Belaich, J.P., and Bayer, E.A. (2001). Design and production of active cellulosome chimeras. Selective incorporation of dockerin-containing enzymes into defined functional complexes. *J Biol Chem* 276**,** 21257-21261. doi: 10.1074/jbc.M102082200.

Giraldo, R. (2010). Amyloid assemblies: protein legos at a crossroads in bottom-up synthetic biology. *Chembiochem* 11**,** 2347-2357. doi: 10.1002/cbic.201000412.

Koch-Schmidt, A.C., Mattiasson, B., and Mosbach, K. (1977). Aspects of microenvironmental compartmentation. An evaluation of the influence of restricted diffusion, exclusion effects, and enzyme proximity on the overall efficiency of the sequential two-enzyme system malate dehydrogenase--citrate synthase in its soluble and immobilized form. *Eur J Biochem* 81**,** 71-78. doi: 10.1111/j.1432-1033.1977.tb11928.x.

Leonard, E., and Koffas, M.A. (2007). Engineering of artificial plant cytochrome P450 enzymes for synthesis of isoflavones by Escherichia coli. *Appl Environ Microbiol* 73**,** 7246-7251. doi: 10.1128/AEM.01411-07.

Mansson, M.O., Siegbahn, N., and Mosbach, K. (1983). Site-to-site directed immobilization of enzymes with bis-NAD analogues. *Proc Natl Acad Sci U S A* 80**,** 1487-1491.

Mingardon, F., Chanal, A., Lopez-Contreras, A.M., Dray, C., Bayer, E.A., and Fierobe, H.P. (2007a). Incorporation of fungal cellulases in bacterial minicellulosomes yields viable, synergistically acting cellulolytic complexes. *Appl Environ Microbiol* 73**,** 3822-3832. doi: 10.1128/AEM.00398-07.

Mingardon, F., Chanal, A., Tardif, C., Bayer, E.A., and Fierobe, H.P. (2007b). Exploration of new geometries in cellulosome-like chimeras. *Appl Environ Microbiol* 73**,** 7138-7149. doi: 10.1128/AEM.01306-07.

Minten, I.J., Claessen, V.I., Blank, K., Rowan, A.E., Nolte, R.J.M., and Cornelissen, J.J.L.M. (2011). Catalytic capsids: the art of confinement. *Chem. Sci.* 2**,** 358-362. doi: 10.1039/c0sc00407c.

Mosbach, K., and Mattiasson, B. (1970). Matrix-bound enzymes. II. Studies on a matrix-bound two-enzyme-system. *Acta Chem Scand* 24**,** 2093-2100. doi: 10.3891/acta.chem.scand.24-2093.

Müller, J., and Niemeyer, C.M. (2008). DNA-directed assembly of artificial multienzyme complexes. *Biochem Biophys Res Commun* 377**,** 62-67. doi: 10.1016/j.bbrc.2008.09.078.

Pinheiro, A.V., Han, D., Shih, W.M., and Yan, H. (2011). Challenges and opportunities for structural DNA nanotechnology. *Nat Nanotechnol* 6**,** 763-772. doi: 10.1038/nnano.2011.187.

Reinke, A.W., Grant, R.A., and Keating, A.E. (2010). A synthetic coiled-coil interactome provides heterospecific modules for molecular engineering. *Journal of the American Chemical Society* 132**,** 6025-6031. doi: 10.1021/ja907617a.

Srere, P.A., Mattiasson, B., and Mosbach, K. (1973). An immobilized three-enzyme system: a model for microenvironmental compartmentation in mitochondria. *Proc Natl Acad Sci U S A* 70**,** 2534-2538.

Terpe, K. (2003). Overview of tag protein fusions: from molecular and biochemical fundamentals to commercial systems. *Appl Microbiol Biotechnol* 60**,** 523-533. doi: 10.1007/s00253-002-1158-6.

Thompson, K.E., Bashor, C.J., Lim, W.A., and Keating, A.E. (2012). SYNZIP protein interaction toolbox: in vitro and in vivo specifications of heterospecific coiled-coil interaction domains. *ACS Synth Biol* 1**,** 118-129. doi: 10.1021/sb200015u.

Wilner, O.I., Weizmann, Y., Gill, R., Lioubashevski, O., Freeman, R., and Willner, I. (2009). Enzyme cascades activated on topologically programmed DNA scaffolds. *Nat Nanotechnol* 4**,** 249-254. doi: 10.1038/nnano.2009.50.

You, C., and Zhang, Y.H. (2014). Annexation of a high-activity enzyme in a synthetic three-enzyme complex greatly decreases the degree of substrate channeling. *ACS Synth Biol* 3**,** 380-386. doi: 10.1021/sb4000993.

Yu, K., Liu, C., Kim, B.G., and Lee, D.Y. (2015). Synthetic fusion protein design and applications. *Biotechnol Adv* 33**,** 155-164. doi: 10.1016/j.biotechadv.2014.11.005.

Yuste, R. (2005). Fluorescence microscopy today. *Nat Methods* 2**,** 902-904. doi: 10.1038/nmeth1205-902.
